# Improving estimates of the burden of severe acute malnutrition and predictions of caseload for programs treating severe acute malnutrition: experiences from Nigeria

**DOI:** 10.1186/s13690-017-0234-4

**Published:** 2017-11-09

**Authors:** Assaye Bulti, André Briend, Nancy M. Dale, Arjan De Wagt, Faraja Chiwile, Stanley Chitekwe, Chris Isokpunwu, Mark Myatt

**Affiliations:** 1 United Nations Children’s Fund (UNICEF), Abuja, Nigeria; 2 University of Tampere School of Medicine and Tampere University Hospital, University of Tampere, Center for Child Health Research, Lääkärinkatu 1, Arvo Building, FI-33014 University of Tampere, Tampere, Finland; 3 Department of Nutrition, Exercise and Sports, Faculty of Science, University of Copenhagen, Rolighedsvej 30, DK-1958 Frederiksberg, Denmark; 4 United Nations Children’s Fund (UNICEF), Nepal Country Office, UN House, Pulchowk, Lalitpur, Kathmandu, Nepal; 5 Department of Family Health, Head of Nutrition/SUN Focal Point, Federal Ministry of Health, Abuja, Nigeria; 6 Brixton Health, Alltgoch Uchaf, Llawryglyn, Powys, Wales, SY17 5RJ UK

**Keywords:** Severe acute malnutrition, Burden, Caseload, Prevalence, Incidence, Nigeria

## Abstract

**Background:**

The burden of severe acute malnutrition (SAM) is estimated using unadjusted prevalence estimates. SAM is an acute condition and many children with SAM will either recover or die within a few weeks. Estimating SAM burden using unadjusted prevalence estimates results in significant underestimation. This has a negative impact on allocation of resources for the prevention and treatment of SAM. A simple method for adjusting prevalence estimates intended to improve the accuracy of burden estimates and caseload predictions has been proposed. This method employs an incidence correction factor. Application of this method using the globally recommended incidence correction factor has led to programs underestimating burden and caseload in some settings.

**Methods:**

A method for estimating a locally appropriate incidence correction factor from prevalence, population size, program caseload, and program coverage was developed and tested using data from the Nigerian national SAM treatment program.

**Results:**

Applying the developed method resulted in errors in caseload prediction of about 10%. This is a considerable improvement upon the current method, which resulted in a 79.5% underestimate. Methods for improving the precision of estimates are proposed.

**Conclusions:**

It is possible to considerably improve predictions of caseload by applying a simple model to data that are readily available to program managers. This implies that more accurate estimates of burden may also be made using the same methods and data.

**Electronic supplementary material:**

The online version of this article (10.1186/s13690-017-0234-4) contains supplementary material, which is available to authorized users.

## Background

A child with severe acute malnutrition (SAM) has a high risk of near term mortality [1, 2]. It has been estimated that SAM affected more than 16 million children globally in 2016 [3]. This figure is based on prevalence estimates from cross-sectional surveys. SAM is an acute condition and many children with SAM will either recover or die within a few weeks. Estimating the number of SAM cases present in a population over a given period of time, the “SAM burden”, using unadjusted prevalence estimates is likely, therefore, to miss many new (incident) cases and significantly underestimate the SAM burden [4]. A recent estimate of the annual global SAM burden that attempts to account for incident cases suggests that 110 million cases per year might be a more accurate estimate [5]. Poor estimates of SAM burden are a problem for program managers at all levels. Underestimation has a negative impact on the prioritization of resource allocation for the prevention and treatment of SAM both globally and locally [6].

Burden is the sum of prevalent cases at the start of a period and incident cases that arise during that period. The number of prevalent cases in a population at a given point in time can be estimated using a combination of a prevalence estimate from a cross-sectional survey and population data. This information is usually already available to program managers. Incidence is more complicated and more expensive to estimate.

**Fig. 1:**
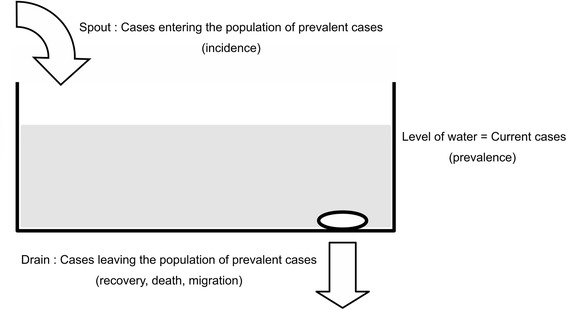
The “bathtub” metaphor for the relationship between incidence and prevalence. The rate at which cases leave the population depends upon the average duration of illness

The relationship between incidence and prevalence is frequently described using a “bathtub” metaphor [7]. In this model the flow of water into the bathtub is analogous to incidence, the level of the water in the bathtub represents prevalence, and the flow of water out of the bathtub through the drain represents recovery and mortality. Incidence in relation to prevalence depends, to a large extent, upon the average duration of illness (see Fig. 1).

The simple relationship between prevalence, incidence, and duration of illness makes it possible to create a simple mathematical model that allows the estimation of burden using prevalence and population estimates together with other data (e.g. program coverage and program caseloads) that will usually be available to program managers.

The Community Management of Acute Malnutrition (CMAM) Forum has proposed a simple method to estimate SAM burden and predict the number of cases that a program will treat over a given planning period [8]. The number of prevalent cases present in a population at the time of a prevalence survey is estimated as the product of prevalence and population size:

$$ Estimated number of prevalent cases= NP $$

where:

$$ N\;\mathrm{is}\  \mathrm{the}\  \mathrm{size}\  \mathrm{of}\  \mathrm{the}\  \mathrm{population}\  \mathrm{of}\  \mathrm{interest} $$

$$ P\;\mathrm{is}\  \mathrm{the}\  \mathrm{prevalence}\  \mathrm{of}\  \mathrm{the}\  \mathrm{condition}\  \mathrm{of}\  \mathrm{interest} $$

The population burden (*B*) consists of both prevalent cases and new (incident) cases that are expected to occur in the program area over a given planning period:

$$ Burden\ (B)= Estimated number of prevalent cases+ Expected number of incident cases $$

The expected number of incident cases can be estimated using:

$$ Expected numer of incident cases= NPK $$

where *K* is a correction factor [9] calculated as:

$$ K=\frac{Duration of planning period}{Average duration of\ a\  disease episode} $$

This allows the population burden (*B*) to be estimated:

$$ B= Estimated number of prevalent cases+ Expected number of incident cases $$

$$ B= NP+ NPK $$

$$ B= NP\left(1+K\right) $$

The population burden (*B*) can be used to predict the number of cases that a program will treat over the planning period (*L*) using an estimate of program coverage (*C*):

$$ Expected program caseload\ (L)= Expected coverage\ (C)\times Population burden\ (B) $$

$$ L= CNP\left(1+K\right) $$

All of the terms in this estimator are subject to uncertainty.

Uncertainty regarding coverage (*C*) and prevalence (*P*) is usually quantifiable and is quantified by confidence intervals or credible intervals on point estimates. The prevalence of severe acute malnutrition (SAM) is often estimated with poor relative precision. For example, the commonly used Standardised Monitoring and Assessment of Relief and Transitions (SMART) prevalence surveys typically have *effective sample sizes* (i.e. the sample size after accounting for survey design effects) between *n* = 300 and *n* = 400 [10]. An effective sample size of *n* = 400 yields an exact 95% confidence interval of [0.55%; 3.24%] on a 1.50% point estimate of SAM prevalence [11]. The relative precision of this estimate is:

$$ Relative precision\ \left(\%\right)=\frac{3.24-0.55}{1.50}\times 100=179.3\% $$

Coverage is typically estimated with a precision of about ± 10% on a 50% estimate [12]. This is a 40% relative precision.

Useful accuracy of population estimates can be achieved by correcting census data to account for population growth and migration. It can often be assumed that the population is estimated with little or no error. This may not, however, be the case in emergencies in which there is considerable and ongoing population movement and / or high levels of mortality.

Caseload (*L*) is a simple count of program admissions. This data is collected and reported on a routine basis and can usually be assumed to be measured with little or no error.

There is considerable uncertainty about the value of the incidence correction factor (*K*). The average duration of an untreated SAM episode that is currently being used globally is 7.5 months. This is based on data from two cohort studies and provides an incidence correction factor (*K*) of 1.6 for a one-year planning period [13]. It was assumed that this value of *K* would apply in all contexts. Governments, United Nations agencies, non-governmental organizations (NGOs), and other SAM treatment program implementing partners have, in the absence of other evidence, been using this value of *K* to estimate the burden and expected caseload and to advocate for resources to treat children with SAM. Reports from SAM treatment programs suggest that the use of *K* = 1.6 has led to programs underestimating caseload in some West African settings. Recent work indicates that a single value of *K* for use globally may not be useful (see Table 1) [6, 14–16].

**Table 1:** Values of incidence correction factors (*K*) found in recent studies^a^

Country	Year(s)	*K* ^b^	SAM case definition(s)^c^	W/H Reference^c^	Data Source(s)	Method	Source
Niger	2010–2013	4.30–9.50	W/H < −3 z-scores or MUAC <115 mm or bilateral pitting edema	WGS	Surveillance system (weekly) Routine program data (weekly) Routine program data (monthly)	Simple mathematical models	Deconinck et al., 2016 [14]
Niger	2006–2007	5.37–11.78	W/H < −3 z-scores or MUAC <115 mm or bilateral pitting edema	WGS	Community cohort (monthly)	Compartmental model to estimate mean duration of SAM episodes	Isanaka et al., 2011 [6]
Mali	2010–2013	2.10–2.50	W/H < −3 z-scores or MUAC <115 mm or bilateral pitting edema	WGS	Community cohort (quarterly) Surveys (occasional)	Simple mathematical models	Isanaka et al., 2016 [15]
Niger	2010–2011	5.00–8.10	W/H < −3 z-scores or bilateral pitting edema	WGS	Community cohort (monthly) Surveys (monthly)	Simple mathematical models	
Burkina Faso	2009–2010	7.30–17.00	MUAC <110 mm (prevalence) MUAC <120 mm (incidence)	WGS	Surveys (annual) Routine program data (monthly)	Simple mathematical models	
Various^d^	2005–2009	11.21	W/H < 70% of median	NCHS	Surveys Caseloads for 5 months after survey	Linear regression	Dale et al., 2017 [16]

^a^A range of methods and data sources were used (surveillance systems, workload returns, cohort studies, repeated cross-sectional surveys, compartmental models, and regression of observed caseload against prevalence) were used to estimate the incidence correction factors (*K*). Refer to the original articles for details

^b^A range of values (i.e. from different methods, data sources, settings, and case-definitions for severe acute malnutrition) is given when available

^c^SAM = severe acute malnutrition, W/H = weight-for-height, MUAC = mid-upper-arm circumference, WGS = World Health Organization child growth standards, NCHS = National Center for Health Statistics child growth reference

^d^24 datasets (surveys and program admissions) from DRC (8), Burundi (2), Somalia (2), Sudan (7), Myanmar (2), and Niger (3). The incidence correction factor (*K*) given in the table is for pooled data assuming coverage (*C*) of 38% (from Rogers et al., 2015). Considerable variation in *K* between settings was observed

Data from the Nigerian Community-based Management of Acute Malnutrition (CMAM) program from 2014 and 2015 are presented in this article. This program started operations in 2009 and has treated between 300 thousand and 500 thousand SAM cases each year. During the course of implementation it was recognized that the use of *K* = 1.6 had led to considerable underestimation of SAM burden and program caseload. Given the public health and security situation in Nigeria it is anticipated that the Nigerian CMAM program will run for many years and accurate estimates of expected caseloads will be required to secure adequate continued funding.

This article presents a method to adjust or calibrate the value of *K* using the population of the program area, the number of program admissions, estimates of program coverage, and estimates of the prevalence of SAM in order to provide more accurate estimates of burden and expected caseload during program implementation. The method is illustrated using data from the Nigerian CMAM program. The revised estimate of *K* may also be useful to predict SAM burden and caseload from prevalence surveys in similar settings.

## Methods

The caseload estimation formula:

$$ L= CNP\left(1+K\right) $$

can be rearranged to find *K* given the other terms:

$$ L= CNP\left(1+K\right) $$

$$ 1+K=\frac{L}{CNP} $$

$$ K=\frac{L}{CNP}-1 $$

A suitable value for *K* can be found by substituting known values for *L*, *C*, *N*, and *P* with *L* being the *observed* program caseload (i.e. the number of admissions).

The method outlined here assumes that both population (*N*) and caseload (*L*) are measured with little or no error although the method can be easily extended to accommodate uncertainty in these terms. The principal sources of uncertainty in this analysis are, therefore, prevalence (*P*) and coverage (*C*). This can lead to considerable uncertainty in their product (*PC*) used in the estimator (Additional file 1).

An approximate 95% confidence interval for the product of two proportions (i.e. prevalence (*P*) and coverage (*C*) in this application):

$$ \widehat{\theta}=P\times C $$

is given by:

$$ \widehat{\theta}\times {e}^{\pm 1.96\times SE\left(\log \widehat{\theta}\right)} $$

where:

$$ SE\left(\log \widehat{\theta}\right)=\sqrt{\frac{1-P}{n_P\times P}+\frac{1-C}{n_C\times C}} $$

and *n*_*P*_ and *n*_*C*_ are the sample sizes used to estimate prevalence (*P*) and coverage (*C*) respectively [17]. This formula is not immediately applicable to the sorts of data likely to be available to program managers because the effective sample sizes used to estimate both prevalence and coverage (*n*_*P*_ and *n*_*C*_) will differ from *reported sample sizes* due to *design effects* introduced by the use of complex samples and / or the use of prior information [12, 18, 19].

Prevalence is usually estimated using surveys employing complex sample designs. The prevalence estimates used in this report were made by combining results from several cross-sectional household surveys that used a two-stage cluster sample design representative at the state level following the SMART methodology [10, 20, 21].

Coverage of CMAM programs is often estimated using spatially stratified samples [12, 22–24]. Semi-Quantitative Evaluation of Access and Coverage (SQUEAC) coverage assessments use a Bayesian beta-binomial conjugate analysis in which the conjugate prior contains information that contributes “pseudo-observations” to the analysis [12, 19].

The effective sample size associated with the estimate of a proportion can be calculated from the reported point estimate (*p*) and its associated upper and lower 95% confidence limits (*UCL* and *LCL*).

Variance (*VAR*) is calculated as:

$$ VAR={\left(\frac{UCL- LCL}{2\times 1.96}\right)}^2 $$

The effective sample size (*n*_*effective*_) is calculated as:

$$ {n}_{effective}=\frac{p\left(1-p\right)}{VAR} $$

rounded to the nearest whole number.

This calculation is performed to find both *n*_*P*_ and *n*_*C*_ before calculating $SE\left(\log \widehat{\theta}\right)$.

We used the approach outlined above to find a suitable value for *K* for the Nigerian CMAM program. Data relating to program admissions (i.e. caseload) in 2014 and 2015 were taken from routine program monitoring reports. Population estimates were made using data from the 2006 Nigerian Census corrected for population growth and migration [25]. Prevalence estimates for SAM were available for 2014 and 2015 [20, 21]. An estimate of program coverage was available from a wide-area Simplified Lot-quality-assurance Evaluation of Access and Coverage (SLEAC) survey completed in early 2014 [12, 26, 27]. The reported coverage from this survey was used for both 2014 and 2015 since it was the only coverage data available. Data were entered and analyzed using Microsoft Excel. This software was used because it is likely to be available and familiar to CMAM program managers. A Microsoft Excel spreadsheet that performs the required calculations is provided as online supporting material. All calculations were checked using the *R* Language and Environment for Statistical Computing version 3.3.3 [28].

The method used to calculate the 95% confidence limits for the product of prevalence and coverage (*PC*) is *approximate*. A less approximate 95% confidence interval (i.e. an interval that contains the true value close to 95% of the time) may be calculated using a bootstrap estimator [29, 30]. Estimates of the incidence correction factor (*K*) were made using a bootstrap estimator for the product of prevalence and coverage (*PC*). A percentile bootstrap estimator with one million replicates for prevalence and coverage drawn from appropriate binomial distributions was used [29]. Data were analyzed using the *R* Language and Environment for Statistical Computing version 3.3.3 [28].

## Results

Table 2 shows the observed and expected (i.e. calculated using *K* = 1.6) caseloads and the revised incidence correction factors (*K*) for 2014 and 2015 together with the data on which the calculations were based. Use of *K* = 1.6 to predict caseload had resulted in gross underestimates in both years. The resulting revised estimates for *K* were *K* = 14.39 (95% CI = 6.64; 30.02) and *K* = 11.66 (95% CI = 5.94; 22.10) for 2014 and 2015 respectively. These estimates were pooled giving *K* = 13.02 (95% CI = 6.80; 19.25). The final two rows of Table 2 show the expected caseloads for 2014 and 2015 using the pooled estimate for *K* and difference between the observed and expected caseloads. Table 3 compares estimates of the incidence correction factor (*K*) calculated using the approximate method and the bootstrap estimator.

**Table 2:** Incidence correction factors for northern Nigeria CMAM program 2014 and 2015 and the data used to calculate them

		Year		
		2014	2015	Data sources
Population	*N*	3,550,827	4,281,700	Nigeria census 2006 corrected for population growth and migration. Population is for children aged between 6 and 59 months in districts in which CMAM services were delivered.
Prevalence^a^	*P*	1.60% (0.50%; 2.71%)	2.01% (0.82%; 3.19%)	Pooled prevalence from state level SMART surveys
Program Coverage^b^	*C*	36.6% (32.3%; 40.9%)	36.6% (32.3%; 40.9%)	Wide-area SLEAC survey
Observed caseload	*L*	320,047	398,676	Routine program monitoring data
Expected caseload (using *K* = 1.6)	*E*_*K* = 1.6_ = *CNP*(1 + *K*)	54,063	81,897	*C*, *N*, and *P* as above (C and P expressed as proportions). Calculations are based on *K* = 1.6
Difference (observed − expected)	*L* − *E*_*K* = 1.6_	265,984	316,779	Calculated as the difference between observed caseload (*L*) and expected caseload (*E*).
Prevalence × Coverage	*PC*	0.59% (0.29%; 1.18%)	0.74% (0.40%; 1.34%)	Calculated (see text)
Incidence correction factor^c^	$K=\frac{L}{PCN}-1$	14.39 (6.64; 30.02)	11.66 (5.94; 22.10)	Calculated (see text)
Expected caseload (using pooled adjusted incidence correction factor)	*E*_*K* = 13.02_ = *CNP*(1 + *K*)	291,527	441,612	*C*, *N*, and *P* as above (C and P expressed as proportions). Calculations are based on *K* = 13.02 (see text).
Difference (observed − expected)	*L* − *E*_*K* = 13.02_	28,520	−42,936	Calculated as the difference between observed caseload (*L*) and expected caseload (*E*_*K = 13.02*_).

^a^Prevalence is for MUAC <115 mm or bilateral pitting edema. This case-definition accounts for c. 98% of all program admissions based on an analysis of routine program monitoring data from two states of northern Nigeria (*n* = 102,245 admissions from January 2010 to December 2013). Prevalence estimates for the states in which the program was operating are reported. This was calculated as the population weighted average of SMART survey results from individual states

^b^Coverage refers to *point coverage* (i.e. the proportion of current SAM cases found by the survey that were enrolled in the CMAM program). Results from a wide-area SLEAC survey from February 2014 are used for both years [Banda et al., 2014]

^c^The formula of the estimator for *K* is rearranged to reflect the fact that PC was calculated prior to use

**Table 3:** Estimates of the incidence correction factor made using two methods to calculate the product of prevalence and coverage

Year	K (approximate)	K (bootstrap)
2014	14.39 (6.64; 30.02)	14.72 (7.73; 40.44)
2105	11.66 (5.94; 22.10)	11.91 (6.17; 27.08)
Pooled	13.02 (6.80; 19.25)	13.32 (6.10; 20.53)

## Discussion

The approach outlined in this document can provide useful estimates of locally appropriate incidence correction factors. Applying the value of *K* estimated for 2014 to the population, prevalence, and coverage data for 2015 yields a predicted caseload of 484,766 cases. This is a 21.6% overestimate of the observed caseload for 2015. Some of this error may have been due to lower than specified coverage during the implementation phase of additional CMAM programming initiated in early 2015 as part of the ongoing emergency response in Northern Nigeria. This degree of error in caseload prediction is a considerable improvement upon the 79.5% underestimate experienced when using *K* = 1.6. Applying the pooled estimate of *K* (i.e. *K* = 13.02) to the population, prevalence, and coverage data yields predicted caseloads of 291,257 cases and 441,612 cases for 2014 and 2015 respectively. These are a 9.0% underestimate and 10.8% overestimate of the true cases for 2014 and 2015. Error are likely to decrease over time as more annual estimates of *K* become available. Not all errors have the same consequences. For example, overestimation may have positive consequences if program coverage is limited by stop-start funding and supply breaks caused by underestimation of burden and / or predicted caseload. Underestimation may lead to an under-resourced program in which program activities essential to achieving and maintaining coverage (e.g. community mobilization, community sensitization, and community-based case-finding activities) are neglected in order to maintain core clinical activities. Underestimation, in some cases, may lead to supply breaks necessitating the temporary closure of programs.

Confidence intervals for the bootstrap estimates of the incidence correction factors are wider than when the approximate method is used. Estimates made using the approximate method are likely to be spuriously precise. The use of approximate methods to calculate confidence intervals is, however, a widely accepted practice for many public health applications. The approximate method has the advantage of being easy to implement using software, such as Microsoft Excel, that is available and familiar to CMAM program managers.

Estimates of the incidence correction factor (*K*) lack precision even when the approximate method is used. For example, the 95% confidence interval for the 2015 estimate of the incidence correction factor (*K*) using the approximate method ranges between *K* = 5.94 and *K* = 22.10. This translates into a 95% confidence interval for the caseload prediction of between about 218 thousand and 728 thousand. This degree of imprecision may limit the utility of the method as a planning tool.

The principal sources of imprecision are in estimates of prevalence and coverage. Improving the precision of estimates of prevalence and / or coverage will improve the precision with which the incidence correction factor (*K*) is estimated.

SAM prevalence is usually estimated with poor relative precision. Relative precision of the prevalence estimates are 138% for the 2014 SAM prevalence estimate and 118% for the 2015 prevalence estimate. The lack of precision in prevalence estimates is due, in part, to the use of sample designs that reduce the effective sample sizes of surveys. It is likely that precision could be improved using, for example, stratified sample designs and larger sample sizes. This would, however, require considerable changes to current practice. Lack of precision is also due to the way that survey data are analyzed. Replacing the classic estimator:

$$ Prevalence=\frac{Number of\  SAM\  cases found in the survey sample}{Survey sample size} $$

with a PROBIT estimator has been demonstrated to reduce the half-width of 95% CIs by about 60% with only small losses of accuracy [31–33]. Slightly Larger gains in precision have been demonstrated using a Bayesian-PROBIT estimator [19, 34]. The advantage of data analytic approaches to improving precision are that they can be applied to data collected with currently used survey methods including historical data at little extra cost.

The precision of the coverage estimate was not an issue in the work reported here because a large stratified sample was used to estimate coverage with good relative precision (i.e. 23.5%). Precision of coverage estimates may, however, be a problem for smaller programs. We investigated this issue using data from 227 SQUEAC coverage assessments of district-level NGO-delivered CMAM programs performed between January 2010 and July 2015 and provided to us by the Coverage Monitoring Network. The median relative precision for coverage estimates between 40% and 60% was 42.6% (IQR = 38.4%; 48.5%). This is an expected result as SQUEAC coverage assessments are usually designed to estimate coverage with this level of precision [12].

The poorer relative precision of SAM prevalence estimates means that efforts to improve the precision of these estimates are likely to yield greater improvements in the precision with which the incidence correction factor (*K*) is estimated than may be achieved by efforts to improve the precision of coverage estimates. This is illustrated in Table 4 using the data from 2015. It is important to note that improvement in the precision of prevalence estimates can be achieved with very little increase in costs but that improvements in the precision of coverage estimates would entail considerable increases in costs.

**Table 4:** Effect of improved precision of SAM prevalence estimates and coverage estimates of the precision of the estimate of the incidence correction factor (*K*) using 2015 data from the Nigerian CMAM program

	Incidence correction factor (*K*)			
Scenario	Point estimate	95% LCL	95% UCL	Relative precision^a^
No change	11.66	5.94	22.10	139.59%
Reduce half-width of 95% CI for prevalence by 60%^b^	11.66	7.72	17.37	82.76%
Reduce half-width of 95% CI for coverage by 60%^c^	11.66	6.00	21.88	136.19%

^a^Relative precision is calculated as $\frac{95\% UCL-95\% LCL}{Point Estimate}\times 100$. Smaller values indicate better precision

^b^This level of improvement is achievable using a PROBIT estimator with existing survey designs and survey data

^c^This level of improvement could only be achieved by a considerable increase in survey sample sizes

### Limitations

A key limitation of the work reported here is that coverage data was not current, particularly for 2015.

A limitation of the method described here is that burden and caseload may be influenced by migration into and out of the program area. Rapid and substantial changes in the population of the program area are likely to affect population size (*N*), prevalence (*P*), and program coverage (*C*). Migration may, therefore, result in grossly inaccurate predictions of burden (*B*) and caseload (*L*) that are based on estimates of population size (*N*), prevalence (*P*), and program coverage (*C*). Monitoring population movements and adjusting burden and caseload predictions may help to address this problem. Adjustment may also require that additional prevalence and coverage surveys be undertaken. In the case of the Nigerian CMAM program there have been reports of SAM cases entering Nigeria from Niger and being admitted to CMAM sites in districts that border Niger. The effect of this on the work reported here is likely to be small since data for the whole country were used. It is important to note that this may have larger effects on burden (*B*) and caseload (*L*) predictions for (e.g.) small NGO-delivered programs operating in border districts.

The assumption that caseload (*L*) is measured with little or no error may also be a limitation. In the case of the Nigerian CMAM program there have been reports from 3 of the 114 districts in which the program is operating of beneficiaries being registered at more than one CMAM site with the assumed intention of receiving additional food and drugs. New CMAM sites were opened in these districts and some of the double registration may have been due to informal transfers between sites. An informal transfer would have been reported as a new admission at the destination site and, some weeks later, as a defaulting patient at the originating site. The effect of this would have been to increase reported caseload (*L*). It seems likely that double registration will have had only a small effect on caseload (*L*) used in the work reported here. This would have caused only a small increase in the estimates for *K* reported here. The covert nature of some double registrations does mean that the magnitude of any increase will always be difficult to quantify.

## Conclusion

The work reported here shows that it is possible to considerably improve predictions of CMAM caseload by applying a simple mathematical model to data that are readily available to program managers. This implies that more accurate predictions of burden may also be made using the same methods and data. The precision of estimates of caseload and burden may be improved by using PROBIT or Bayesian-PROBIT estimators of SAM prevalence.

The implication of this study, and of similar reports based on a variety of approaches (see Table 1), is that the current estimates of SAM burden are likely to be gross underestimates. Applying the pooled incidence correction factor found in this study to the 16 million estimate made using prevalence data yields an estimated global SAM burden of 208 (95% CI = 109; 308) million cases annually. It seems unlikely, however, that the incidence correction factor estimated for the Nigerian CMAM program will be globally applicable. Local estimates of *K* will be needed to make local prediction of burden and caseload. These local estimates of *K* could be applied to local estimates of prevalence and population with the results summed in order to estimate global SAM burden.

Given the public health importance of having reliable estimates of burden and caseload and the uncertainties of this approach based on program data, a confirmation of estimates of *K* using direct estimates of incidence from continuous monitoring of open cohorts and surveillance systems in similar settings may be warranted. Comparison with other indirect methods may also prove useful.

## Additional file

Additional file 1: Caseload method. (XLSX 36 kb)
